# Techno-economic evaluation of 2^nd ^generation bioethanol production from sugar cane bagasse and leaves integrated with the sugar-based ethanol process

**DOI:** 10.1186/1754-6834-5-22

**Published:** 2012-04-13

**Authors:** Stefano Macrelli, Johan Mogensen, Guido Zacchi

**Affiliations:** 1 Department of Chemical Engineering - Lund University, P.O. Box 124, S-22100 Lund, Sweden; 2 Novozymes A/S, Krogshoejvej 36, Bagsvaerd DK-2880, Denmark

**Keywords:** Bioethanol, Second-generation ethanol, Advanced biofuel, Lignocellulose, Sugar cane, Bagasse, Simulation, Techno-economic evaluation, Production cost, Minimum ethanol selling price

## Abstract

**Background:**

Bioethanol produced from the lignocellulosic fractions of sugar cane (bagasse and leaves), i.e. second generation (2G) bioethanol, has a promising market potential as an automotive fuel; however, the process is still under investigation on pilot/demonstration scale. From a process perspective, improvements in plant design can lower the production cost, providing better profitability and competitiveness if the conversion of the whole sugar cane is considered. Simulations have been performed with AspenPlus to investigate how process integration can affect the minimum ethanol selling price of this 2G process (MESP-2G), as well as improve the plant energy efficiency. This is achieved by integrating the well-established sucrose-to-bioethanol process with the enzymatic process for lignocellulosic materials. Bagasse and leaves were steam pretreated using H_3_PO_4 _as catalyst and separately hydrolysed and fermented.

**Results:**

The addition of a steam dryer, doubling of the enzyme dosage in enzymatic hydrolysis, including leaves as raw material in the 2G process, heat integration and the use of more energy-efficient equipment led to a 37 % reduction in MESP-2G compared to the Base case. Modelling showed that the MESP for 2G ethanol was 0.97 US$/L, while in the future it could be reduced to 0.78 US$/L. In this case the overall production cost of 1G + 2G ethanol would be about 0.40 US$/L with an output of 102 L/ton dry sugar cane including 50 % leaves. Sensitivity analysis of the future scenario showed that a 50 % decrease in the cost of enzymes, electricity or leaves would lower the MESP-2G by about 20%, 10% and 4.5%, respectively.

**Conclusions:**

According to the simulations, the production of 2G bioethanol from sugar cane bagasse and leaves in Brazil is already competitive (without subsidies) with 1G starch-based bioethanol production in Europe. Moreover 2G bioethanol could be produced at a lower cost if subsidies were used to compensate for the opportunity cost from the sale of excess electricity and if the cost of enzymes continues to fall.

## Background

Currently in Brazil, the production of sugar cane bioethanol is based entirely on the fermentation of sugar juice from sugar cane and/or molasses in autonomous distilleries (39% of the cases) and in plants associated with sugar mills (61%) [1]. This technology has been in commercial use for the past 30 years and can be considered to be mature as the cost of feedstock accounts for a major part of the production cost [2], around 60-70% [3,4]. Compared to other crops used for bioethanol production from sugar and starch, i.e. first-generation (1G) bioethanol, bioethanol from sugar cane is claimed to have the lowest production cost worldwide [5]. The low cost of Brazilian 1G bioethanol can be explained by a combination of favourable conditions such as the photosynthetic rate of the sugar cane crop per hectare, the meteo-climatic conditions and a renewable energy ratio close to 10, denoting an efficient life cycle from cultivation to processing included [6]. The 2010/2011 sugar cane harvest in Brazil yielded 625 Mton sugar cane (SC) leading to the production of hydrous and anhydrous ethanol of 19.6 and 8.1 billion litres, respectively [7]. Recently, the high sugar prices, the ethanol export and the high internal demand for hydrous ethanol for flex-fuel cars caused the Brazilian Federal Government to reduce the amount of anhydrous ethanol blended in gasoline from 25% to 20%, and to direct it towards hydrous ethanol [8]. In 2011 Brazil was still a net exporter of ethanol even if since 2008 export has decreased 3.4-fold reaching the value 2 billion litres [9]. Also for this reason, it was recently announced that incentives for ethanol production will be available for new autonomous distilleries [10]. Furthermore, the conventional sucrose-to-bioethanol process yields not only ethanol and crystallized sugar, but also bagasse, a lignocellulosic residue with an interesting higher heating value, some of which can be used in boilers to produce the heat and electricity needed to run the plant.

The view of bagasse has changed throughout the years, due to technological breakthroughs, investment opportunities and revenue margin. At the beginning of the Pro-álcool project in the 1970s, bagasse was considered a fluffy waste which was eliminated in low-efficiency 22- bar boilers. As a result of deregulation of the electricity market and the increase in the price of electricity due to the 2001 energy crisis [11], bagasse has come to be regarded as the perfect solid fuel for bioelectricity generation, and for the past decade it has been combusted in more efficient and expensive boilers [12]. Nowadays, bagasse is also generally recognized as a very promising feedstock for cellulosic ethanol production, i.e. second-generation (2G) bioethanol, and it is also expected that biofuel produced in this way will have less impact on the environment. However, the production cost of 2G bioethanol is still rather high, irrespective of the lignocellulosic feedstock used, and the development of a commercially competitive process for 2G technology poses a challenge [11,13,14], although the integration with 1G production could greatly facilitate this development, as will be discussed in the present study.

Sugar cane leaves and tops, often called trash, constitute the residues of mechanical harvesting, and are suitable as raw material for 2G bioethanol production because of their lignocellulosic nature. The amount of trash that can be removed from the field without affecting the nutritional properties of the soil or pest and weed control is generally considered to be about 50% by weight [15], although it is strongly dependent on the location and weather. Recent estimates show that up to 66% of the trash can be used for industrial processes, if only that amount is removed as dry leaves [16]. For this reason, the term leaves will be used instead of trash since the material removed from the field is mainly composed of leaves.

Generally, the main process bottlenecks in 2G ethanol production, such as the low yield from the conversion of lignocellulose into fermentable sugars and the downstream processing, account for a significant proportion of the production cost [14]. Several techniques for pretreating and hydrolysing the material, as well as process configurations, have been investigated with the aim of remedying the low ethanol productivity and high costs [17].

Among the viable processes, steam pretreatment followed by enzymatic hydrolysis is one of the most promising approaches for ethanol production from lignocellulose [18], and this configuration was adopted in the present study for the 2G ethanol production from sugar cane bagasse and leaves using separate hydrolysis and fermentation (SHF). Other techno-economic studies on ethanol production based on more or less the same process concept have been performed previously for various materials, e.g., switch grass [19], tall fescue [20], hardwood [21], softwood [22,23], straw [24], poplar [25], salix [23], corn stover [23] and sugar cane bagasse [26-28].

Process design and optimization greatly affect the production cost. The choice between simultaneous saccharification and fermentation and SHF, which have different advantages and disadvantages regarding their effects on downstream processing [29]; boiler pressure, which affects the electricity production efficiency [30]; mechanical recompression in evaporation, which reduces the steam demand [31,32]; thermally integrated distillation, which also reduces energy demand as discussed by Dias [33]; as well as other integration options has been investigated in various studies [21,34,35].

Apart from technical hurdles and the price of petrol, the question of feedstock competitiveness must be addressed at international and local levels. The global price of sugar and the local bioelectricity price may affect 1G and 2G ethanol production, respectively. Although there is no direct competition with food security and supply, 2G ethanol from sugar cane suffers from a higher production cost due to the rise in opportunity cost caused by the rapidly growing bioelectricity market in Brazil. Today, greater profit may be achieved by burning bagasse and leaves than by producing 2G ethanol due to a lack of cheap fuel to complement hydropower in the dry season. However, future development of the 2G ethanol process using leaves and bagasse is recognized to be profitable, even when a high surplus of electricity (184 kWh/ton SC) is sold [36]. In addition high sugar prices may reduce the gap between the 2G and 1G ethanol production costs. Furthermore the goal of more competitive 2G ethanol production can also be achieved by integrating the 1G and 2G technologies in a plant sharing energy and material streams in unit operations in order to exploit synergies. It is also important to utilize the whole material for the production of by-products, as this has been shown to have considerable impact on the economic feasibility [14,34].

In the present study, a techno-economic evaluation of integrated 1G and 2G ethanol production from sugar cane has been performed for fifteen scenarios. In each scenario several operating conditions were implemented in the flowsheeting models, based on data obtained experimentally within the CaneBioFuel Project ^# ^[37], and different process layouts were designed to optimize the ethanol production based on these data. The final results of the CaneBioFuel Project regarding the technical design and economic assessment are presented, summarizing the feasibility of the completely integrated production process, considering both overall energy efficiency and the ethanol production cost, for each scenario.

## Results and discussion

The integration of 1G and 2G ethanol production was carried out sequentially by first investigating various process parameters, e.g. water insoluble solids (WIS), enzyme dosage and residence time in the enzymatic hydrolysis (EH), and then choosing the best conditions to evaluate the effect of various levels of integration. Each scenario was assessed by calculating the energy efficiency, and the running and capital costs, all of which have a significant effect on the MESP-2G ethanol. Excluding the Reference scenario, fourteen scenarios of the combined 1G + 2G process (A-L2) were simulated according to the EH operating conditions and process configurations summarized in Table 1. A combination of enzyme dosage (highlow) and EH residence time (72 h-48 h) were investigated in the model for Scenarios B, C, D, E in order to identify the case having the lowest MESP-2G. The resulting scenario was then used as the starting point for the evaluation of leaves addition (Scenario F) and heat integration (Scenario G). Further process integration options were investigated to assess the impact of a common distillation unit (Scenario I) and multiple-effect evaporation unit (Scenario J) between the 1G process and 2G process. Leaves addition together with integration options was also evaluated with respect to WIS (7%, 14%) in Scenarios H, K1, K2. The L1 and L2 Scenarios represented an expected future improvement at higher WIS content (16%) without and with leaves addition, respectively.

**Table 1:** Summary of the conditions for the 1G + 2G scenarios investigated (A-L2)

Parameter							Scenario							
	**A**	**B**	**C**	**D**	**E**	**F**	**G**	**H**	**I**	**J**	**K1**	**K2**	**L1**	**L2**

1G heat integration	-	-	-	-	-	-	YES	YES	YES	YES	YES	YES	YES	YES

1G+2G stream integration	-	-	-	-	-	-	-	-	Distill.	Evap.	Evap.	Evap.	**Future**	**Future**

Leaves	-	-	-	-	-	YES	-	YES	-	-	YES	YES	-	YES

**Enzymatic hydrolysis**														

WIS (%) ^a^	7	7	7	7	7	7|7	7	7|14	7	7	7|14	7|7	16	16|16

Enzyme dosage	low	low	low	high ^b^	high ^b^	high ^b^	high ^b^	high ^b^	high ^b^	high ^b^	high ^b^	high ^b^	low	low

Hydrolysis time (h)	72	72	48	72	48	72	72	72	72	72	72	72	72	72

Glucose yield from EH, (%)^a^	47	47	42	73	66	73|96	73	73|76	73	73	73|76	73|96	47	47|68

^a ^When two values are given the first is for bagasse and the second for leaves.

^b ^The high enzyme loading is twice the low enzyme loading.

### Autonomous distillery - reference scenario

**Figure 1:**
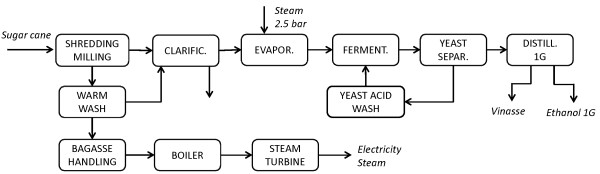
**Schematic flowsheet of the 1G autonomous distillery chosen as the Reference scenario**. This scenario represents the existing configuration for the 1G ethanol production.

First, a Reference scenario was simulated for the 1G process (Figure 1), i.e. the autonomous distillery as it is today, to illustrate the differences in cost and process performance between the existing 1G facility and the proposed integrated 1G + 2G plant. Model results for the autonomous distillery are presented in Table 2 regarding the main streams. The total capital cost is 117 million US$ including the combined heat and power (CHP) plant, comprising the total direct field cost (74 million US$), the indirect field cost (11 million US$) and the total non-field cost (32 million US$).

**Table 2:** Results for the 1G autonomous distillery (Reference scenario)

Parameter	Value	Unit
Sugar cane as raw material	165	dry ton/h

Fresh water required	310	m^3^/h

Ethanol produced	46	m^3^/h

Vinasse produced	370	m^3^/h

Bagasse to boiler	74	dry ton/h

Electricity produced	77	MW_el_

Live steam, 2.5 bar, for evaporators	210	ton/h

Steam, 1.7 bar, required for distillation	160	ton/h

Steam, 1.7 bar, recovered after evaporation	170	ton/h

Total capital cost (including CHP)	117	million US$

Since sugar cane bagasse is used as the only feedstock in the Reference scenario, the electricity opportunity cost is accounted for only for bagasse, even when leaves were added in the integrated process scenarios.

### Scenario A - the base case

**Figure 2:**
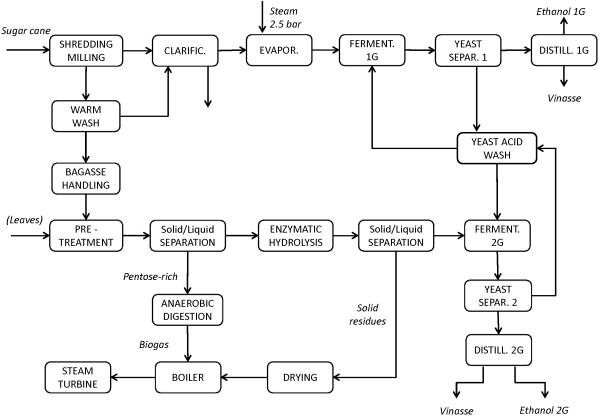
**Schematic flowsheet for the combined 1G + 2G process**. This flowsheet represents Scenarios A, B, C, D, E, F, and is obtained combining the Reference scenario 1G process with the stand-alone 2G process. The flowsheet is also valid for Scenario G and H where the single-effect evaporation unit is replaced with five-effect unit. The dryer was not included in Scenario A, but was included in all other scenarios. Leaves were used in Scenarios F and H.

The Base case (Scenario A) represents the combined 1G + 2G plant, to evaluate the effects on the 2G process of changing the operating conditions and level of process integration between 1G and 2G ethanol production, without mixing the material streams. The schematic flowsheet for the Base case is shown in Figure 2. Sugar cane bagasse is supplied to the 2G process, and the solid residues from EH are burnt in the CHP plant together with the biogas produced from the pentose-rich stream after filtration of the pretreated material. In this case, there is no integration of heat or streams between the 1G and 2G processes. The 2G facility can be seen as an annexed but stand-alone plant. EH is run at 7% WIS for 72 hours with a low enzyme dosage. The EH residues are sent to the CHP plant for steam and electricity production, but the energy obtained is not enough for self-sufficiency of the whole plant. For this reason, 25.4% of the fresh bagasse flow is rerouted to the CHP plant to meet the energy requirements. The overall energy efficiency is 59.2%. The minimum ethanol selling price (MESP) for 1G ethanol from the Reference case, the 1G + 2G MESP for Scenario A and MESP for 2G ethanol for Scenario A are presented in Table 3, which also shows the contribution to the 2G cost from each cost item.

**Table 3:** Cost items and MESP for the Reference scenario (1G ethanol) and Scenario A (Base case)

	Reference scenario (1G ethanol)	Scenario A (1G+2G ethanol)	2G ethanol ^a ^for Scenario A
Ethanol, L/dry ton SC	284	329	45

Power production, kWh/dry ton SC	364	185	-179

**Cost items, US$/L**			

Sugar cane	0.230	0.198	0.000

Enzymes	0.000	0.045	0.341

Acid	0.000	0.005	0.080

Base	0.003	0.003	0.020

Water consumption	0.003	0.008	0.045

Other raw materials	0.008	0.005	0.000

Labour, maintenance, insurance	0.040	0.055	0.148

Electricity export/opportunity cost	-0.111	-0.050	0.357

Vinasse sales	0.000	0.000	-0.003

Capital cost	0.095	0.156	0.560

**Minimum Ethanol Selling Price**	0.264	0.428	1.548

^a ^*2G ethanol and power production are obtained as the difference of Scenario A value and the Reference scenario (1G) value. The cost items for 2G ethanol are calculated according to Equation 1*.

An overview of costs for the simulated scenarios for 1G + 2G process can be found in Table 4 where the total investment cost for each scenario is also included together with the total ethanol production cost, the energy efficiency and the electricity surplus.

**Table 4:** 2G cost items and MESP-2G, 1G + 2G MESP and other economic and energy parameters for all scenarios

Scenario	A	B	C	D	E	F ^a^	G	I	J	H ^a^	K1 ^a^	K2 ^a^	L1 ^a^	L2 ^a^
**2G ethanol, L/ton-dSC**	45	57	53	76	76	151	95	95	95	136	136	151	61	102

**2G cost items, US$/L^b^**														

**Enzymes**	0.34	0.33	0.36	0.42	0.47	0.38	0.42	0.42	0.42	0.41	0.41	0.38	0.31	0.28

**Acid**	0.08	0.08	0.09	0.05	0.06	0.05	0.05	0.05	0.05	0.05	0.06	0.05	0.08	0.07

**Base**	0.02	0.01	0.01	0.01	0.01	0.01	0.01	0.01	0.01	0.02	0.02	0.01	0.02	0.02

**Water consumption**	0.04	0.04	0.04	0.03	0.03	0.02	0.03	0.03	0.02	0.02	0.02	0.02	0.02	0.01

**Leaves**	0.00	0.00	0.00	0.00	0.00	0.05	0.00	0.00	0.00	0.05	0.05	0.05	0.00	0.07

**Labour, maintenance, insurance**	0.15	0.12	0.13	0.09	0.08	0.07	0.10	0.09	0.09	0.09	0.08	0.08	0.06	0.06

**Electricity export/opportunity cost**	0.36	0.31	0.32	0.25	0.27	0.12	0.20	0.19	0.20	0.06	0.08	0.10	0.18	0.01

**Vinasse sales**	0.00	0.00	0.00	0.00	0.00	0.00	0.00	0.00	0.00	0.00	0.00	0.00	0.00	0.00

**Capital cost**	0.56	0.46	0.49	0.34	0.33	0.28	0.36	0.34	0.33	0.34	0.31	0.31	0.22	0.25

**MESP-2G**	**1.55**	**1.36**	**1.45**	**1.19**	**1.25**	**0.97**	**1.16**	**1.12**	**1.11**	**1.05**	**1.02**	**0.99**	**0.88**	**0.78**

**1G+2G MESP**	0.43	0.44	0.45	0.46	0.47	0.51	0.49	0.48	0.48	0.50	0.51	0.52	0.38	0.40

**Internal Rate of Return (%) ^c^**	15.7	15.4	15.0	14.5	14.1	11.5	12.7	11.5	13.4	13.6	11.2	11.4	21.5	18.8

**Total capital cost (*million US$*)**	232	236	234	237	228	299	272	260	259	331	311	334	181	234

**Energy parameters**														

**Plant energy efficiency**	59.2	61.4	61.4	62.0	61.4	58.9	64.7	64.8	64.9	62.6	61.5	60.5	66.6	63.3

**Electricity surplus (kWh/ton SC)**	56	50	52	43	41	50	46	48	46	80	74	60	71	106

^a ^*In this scenario sugar cane leaves were added for the 2G ethanol production*

^b ^*Cost items for the 2G are calculated according Equation 1 and MESP-2G is the sum of these items*

^c ^*The internal rate or return (IRR) is calculated considering the hydrous ethanol selling price of 0.53US$/L, averaged for the years 2008-2011 in the São Paulo State. For the 1G process the IRR is 32.1%*

### Scenario B

Scenario B consists of the Base case (Scenario A) with a steam dryer for the EH solid residues to increase the lower heating value of the fuel by the removal of water, from 35 wt- % to 80 wt-% dry matter (DM). The steam dryer was assumed to work at 20 bar with superheated steam as drying medium. The effect of this in terms of energy is that more heat is available for steam and electricity production due to the reduction in heat loss by water vapour leaving with the flue gases. Therefore, more bagasse is available for ethanol production, but still not the full amount (about 94%). The water removal efficiency of the dryer is better than 85%, calculated from the energy recovery in the secondary steam. The steam dryer increases the heat use by 3.5% resulting in a process energy efficiency of 61.4%. From the cost point of view, the MESP-2G is lower than in Scenario A, indicating that including a dryer paid off. The drying unit was therefore included in all the following scenarios due to the beneficial effects in terms of energy and cost.

Scenario B was then varied, mainly by changing the operating conditions in the EH step such as the enzyme dosage (Scenarios D, E) and the residence time (Scenarios C, E).

### Scenario C

The residence time in EH is a key factor that affects the conversion yield and capital cost directly, and the energy and ethanol production cost indirectly. A short residence time results in both a reduction in sugar yield and a reduction in the capital cost for the EH tanks. In Scenario C the residence time was shortened by 24 h compared with Scenario B, i.e. from 72 to 48 hours. It was still necessary to use a fraction of the bagasse (2%) for combined heat and power production. The main effect was a reduction in the ethanol production, from 57 to 53 L/ton-dry SC (ton-dSC), while there was no variation from the energy efficiency point of view (61.4%). However, the MESP-2G for Scenario C is definitely higher than for Scenario B, so the savings in the capital cost of EH did not balance the decrease in revenue from selling ethanol at the enzyme loading investigated.

### Scenario D

Enzyme loading was changed in the various scenarios to assess the impact of enzyme cost per litre of ethanol produced. In Scenario D the enzyme dosage was double that in Scenario B to investigate if a higher sugar yield and concentration could compensate for the increased cost of enzymes. No other modifications were applied to the flowsheet. As the amount of ethanol produced per ton of bagasse increased, less solid residue was available for CHP generation, and 16.3% of the total bagasse had to be used to ensure self-sufficiency in steam. Due to the higher EH yield, the final ethanol concentration prior to distillation also increased, reducing the energy demand as well as the capital cost for the distillation stage. This is also confirmed by the increase in energy efficiency for Scenario D to 62.0%. Although the total ethanol produced increased by exactly 1/3, the equipment cost did not increase correspondingly. However, the most positive effect of doubling the enzyme dosage was the decrease in the MESP-2G from 1.36 to 1.20 US$/L ethanol.

### Scenario E

A similar comparison was performed between Scenarios C and B to determine whether lowering the residence time had positive effects on the MESP-2G, but at higher enzyme loadings in EH. In Scenario E the enzyme dosage was the same as in Scenario D, while the EH residence time was 48 h instead of 72 h. The amount of bagasse required for CHP production in this scenario was 9.8% of the total amount, so the amount of bagasse available for 2G ethanol was 90.2%. Also in this case, a decrease in residence time did not pay off in spite of higher enzyme loading, and the MESP-2G increased compared to that for Scenario D.

**Figure 3:**
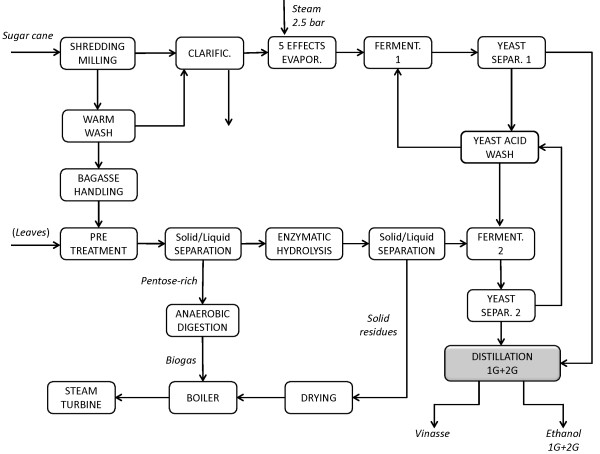
**Schematic flowsheet for Scenario I**. The distillation of 1G and 2G ethanol is performed in the same unit.

**Figure 4:**
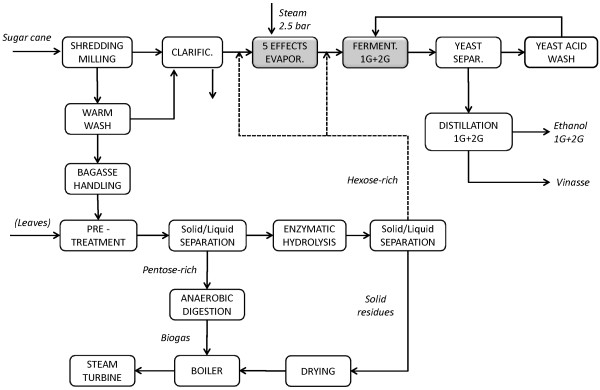
**Schematic flowsheet for Scenarios J, K1 and K2**. The hexose-rich streams from 1G and 2G process are mixed, concentrated by five-effect evaporation and fermented together. Leaves are used in cases K1 and K2.

Among Scenarios A to E, Scenario D showed the lowest MESP-2G, and it was therefore used as the basis for the addition of leaves (Scenario F) and for heat integration of the combined 1G + 2G process (Scenario G). Stream integration was simulated by combining the distillation streams from the 1G and 2G processes in the same unit (Scenario I, Figure 3) and by mixing the sugar streams from 1G and 2G in the evaporation step (Scenario J, Figure 4).

### Scenario G

The first step towards integration involves the heat use in the 1G process. Heat exchangers were employed to recover and share energy flows between unit operations according to the minimum temperature approach for the optimization of the combined 1G + 2G heat network. The single-effect evaporation used in the autonomous distillery was replaced by five-effect evaporation. The live steam used in the first evaporator is superheated to 144°C at 2.5 bar, and the five flash stages are performed at a pressure allowing a change in temperature of at least of 12°C over each evaporator. The energy in the condensate streams is recovered by heat exchanging, to heat up the feed before the first evaporator. The configuration of 1G distillation was also updated to that used for 2G distillation, i.e. heat integrated with two strippers and one rectifier, including preheating of the feed streams using the bottom streams from the corresponding distillation columns. The secondary steam from distillation, evaporation and drying units was reused.

The heat requirement in Scenario G is 130 MW, i.e. 27% lower than in Scenario D (179 MW) which is non-integrated. As a matter of fact, the energy efficiency reached 64.7%. The amount of steam saved allowed the use of all the bagasse for ethanol production, which increased from 76 L/ton-dSC in Scenario D to 95 L/ton-dSC in Scenario G. Consequently, the MESP-2G also decreased, from 1.20 to 1.16 US$/L.

### Scenario I

Scenario I (see Figure 3) is the same as Scenario G except that one common distillation system was used for both 1G and 2G fermentation broths. Since the capital cost is generally lower for a single unit than two smaller units having the equivalent capacity, the main purpose was to verify whether the heat demand arising from mixing the two streams, giving an ethanol concentration of 3.5 wt-%, was lower than for the distillation of the two separate streams with ethanol concentrations of 7 wt-% and 1.4 wt-%. The steam requirement for the joint distillation unit corresponds to a net heat power demand of 68 MW, while it was 72 MW in Scenario G. The MESP-2G in Scenario I is also lower than in Scenario G, demonstrating that using a single distillation unit was beneficial to both the capital cost and heat power demand.

### Scenario J

Another way to integrate streams from 1G and 2G earlier in the process is to mix the sugar streams in the evaporation stage. There are many mixing options, but the one selected from among those investigated (data not presented) consists of blending part of the liquid after EH of bagasse with the sugar juice from cane before and after evaporation in order to obtain an intermediate concentration of 25 wt-% sucrose and a final total concentration of mixed sugars of 8.5 wt-%, as shown in Figure 4. After joint fermentation the ethanol concentration in the feed to the single distillation plant was about 4.2 wt-%. It was thought that the removal of water before distillation with the five-effect evaporator would be beneficial, but the results indicate that Scenarios G and J are almost equivalent in terms of energy efficiency, capital cost and MESP-2G.

The addition of leaves was also evaluated in the integrated Scenarios G and J, resulting in Scenarios H and K1, respectively, where EH of leaves was performed at 14% WIS. In Scenario K2 the EH of leaves was performed at 7% WIS, which is the only difference compared with Scenario K1 (Figure 4).

### Scenarios F and K2

In Scenarios F and K2 leaves are added at an amount corresponding to 50 % of the total amount of bagasse based on dry weight to supplement 2G ethanol production. Pretreatment is carried out in three reactor units working in parallel, two for bagasse and one for leaves. The enzyme dosage and WIS used for EH of the leaves are the same as for bagasse in Scenario D (double enzyme load, 7% WIS). Both Scenarios F and K2 showed the highest 2G ethanol production (151 L/ton-dSC) and yield.

Despite the fact that Scenario F includes no integration (of heat or stream, except for reuse of the secondary steam from the evaporators), the MESP-2G obtained, 0.97 US$/L, is the lowest of all the scenarios investigated. The high level of integration of Scenario K2 (heat and streams), already described in Scenario J, has a greater effect on the energy savings than Scenario F (5 MW). However, the capital cost in Scenario K2 increased to 334 million US$, compared with 299 million US$ in Scenario F. This negative impact on the total production cost for Scenario K2 is greater than the positive effect of the revenue obtained from the 16% increase in the amount of electricity produced, resulting in a slightly higher MESP-2G of 0.99 US$/L.

### Scenarios H and K1

Scenarios H and K1 were modelled as Scenarios G and J respectively, but including leaves in the 2G production. This resulted in a net increase in ethanol production of 41 L/ton-dSC compared with the corresponding scenarios without leaves, obtaining a total of 136 L/tondSC of 2G ethanol. There is no streams integration between 1G and 2G ethanol production in Scenario H while Scenario K1 is fully integrated (heat and streams). In the latter case, integration allows a lower ethanol production cost resulting in a MESP-2G of 1.02 US$/L, compared to 1.05 US$/L in Scenario H. This is mainly due to the lower capital cost in Scenario K1. The enzymatic hydrolysis of the leaves is assumed to be performed at 14% WIS in Scenarios H and K1, which is twice that in Scenarios F and K2. Consequently, the sugar yield from EH falls by 20% due to the difficulty in hydrolysing the material at higher WIS, therefore leaving more EH solid residues for CHP production. For this reason, the electricity export opportunity cost decreases compared with Scenarios F and K2, but the decrease in net ethanol production, by 15 L/ton-dSC, causes an increase in MESP-2G. From an economic point of view it is better to perform the EH of leaves at 7% WIS than 14% WIS.

### Scenarios L1 and L2

**Figure 5:**
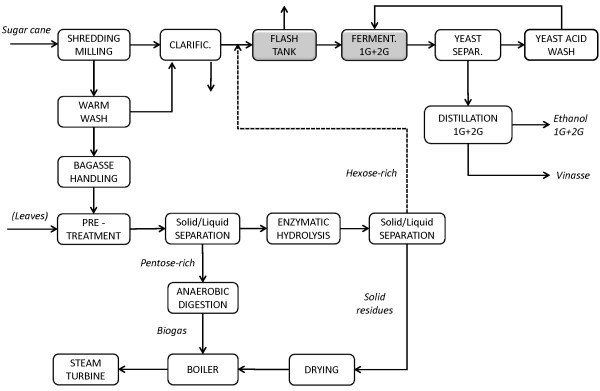
**Schematic flowsheet for future Scenarios L1 and L2**. Only a flash unit is needed to achieve the desired concentration before the combined fermentation of 1G and 2G. Leaves are added in Scenario L2.

Scenarios L1 and L2, shown in Figure 5, represent the expected improvements in EH technology in the near future, at higher WIS, i.e. not based on experimental data but on the extrapolation of the glucose yields from 14 to 16% WIS and assuming that the cost of enzymes is half of those in Scenarios D-K2. This reduction in cost was modelled as half the enzyme dosage. The feedstock for Scenario L1 is bagasse only, while in scenario L2 leaves are added for 2G ethanol. In this case, a conservative EH yield was assumed for the leaves, although experimental results demonstrated better hydrolysis of leaves than bagasse (data not shown). The high WIS content in EH results in a sugar concentration of 8.5 wt-%, which suggests that the five-effect evaporation step could be avoided and replaced by a simple flash tank operating at 65°C. The energy efficiency is the highest of all the scenarios simulated (66.6%). Furthermore, the 2G ethanol production costs were the lowest, with an MESP-2G of 0.88 US$/L in Scenario L1 using only bagasse, and 0.78 US$/L in Scenario L2 using both bagasse and leaves. However, is should be noted that the amount of 2G ethanol produced is greatly reduced, due to the low EH yield, to 61 and 102 L/ton-dSC for Scenarios L1 and L2, respectively, i.e. about 33% lower than the maximum obtained in this study.

The various scenarios simulated in this work give an indication of how a future process for the integration of 2G and 1G ethanol production from sugar cane could be designed in order to achieve the lowest 2G ethanol production cost. However, the number of ways in which heat and streams can be integrated is significantly larger than the number investigated in this project, and we are convinced that it is possible to further reduce the production cost in the future with other integration conditions.

### Overall ethanol production (1G + 2G) MESP

Table 4 shows the overall MESP from both 1G and 2G processes. The MESP-2G and the ethanol volumetric production per ton sugar cane directly influence the MESP for the overall ethanol production. The lowest 1G + 2G MESP was obtained for Scenario L1 although the lowest MESP-2G was found for Scenario L2 (0.78 US$/L). This depends on the higher 2G ethanol volume produced in Scenario L2 (102 L/ton-dSC) compared with Scenario L1 (61 L/ton dSC), which gives a slightly higher average ethanol production cost. In Scenarios F and K2, where the 2G ethanol contributes with an additional 50% to the ethanol production volume, i.e. 151 L/ton-dSC, the 1G + 2G MESP is the highest among all cases and about 0.52 US$/L, which however still can compete with most European starch-based MESPs. Although the economic feasibility is achieved, in terms of profitability the 1G + 2G ethanol production in all the scenarios simulated in this study cannot compete with the 1G ethanol when the ethanol selling price is 0.53 US$/L, due to the lower IRR for the scenarios compared to the 1G process (IRR = 32.1%), as shown in Table 4. This price is the average ethanol selling price for hydrous ethanol calculated over the year 2008-2011 in the São Paulo State [38]. Despite the lower profit compared to 1G ethanol, the 1G + 2G ethanol production is economic feasible even today for all the cases investigated with no subsidies if the ethanol selling price is higher than 0.52 US$/L.

### Sensitivity analysis

**Figure 6:**
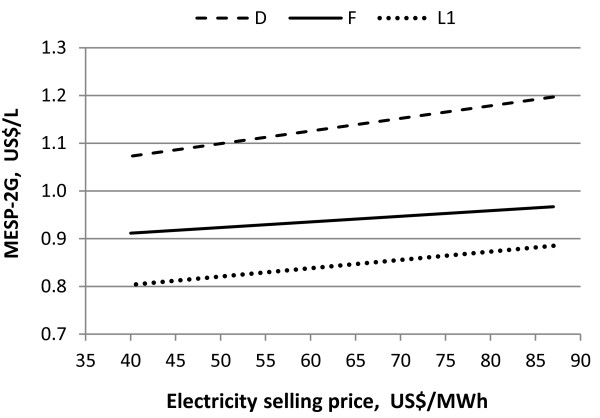
**Influence of the electricity selling price on the MESP-2G for Scenarios D, F and L1**.

The calculated MESP-2G is very sensitive to the electricity selling price and to the cost of leaves and enzyme, thus an analysis of these variables was performed to investigate the impact on the MESP-2G in the most relevant scenarios. Figure 6 shows the effect of the electricity selling price on the MESP-2G for Scenarios D, F and L1. A decrease in electricity price by 50% reduces the MESP-2G by more than 10% in all cases. However, even if the electricity price is set to zero the MESP-2G would not be reduced to the MESP of 1G ethanol, which is calculated to be 0.38 US$/L when no income is considered for electricity.

**Figure 7:**
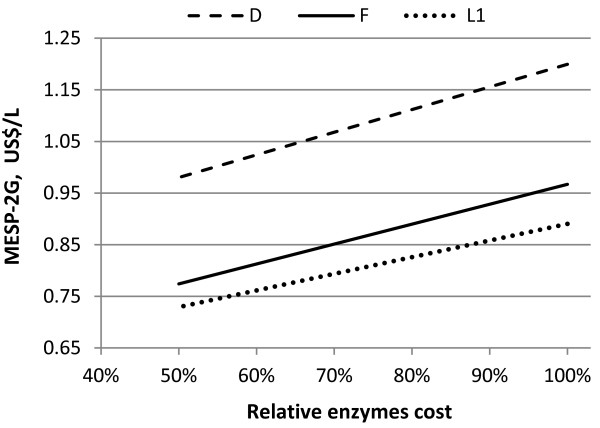
**Influence of enzyme cost on the MESP-2G for Scenarios D, F and L1**.

The impact of enzyme cost on the MESP-2G in Scenarios D, F and L1 is shown in Figure 7. A decrease in enzyme cost of 50% lowers the MESP-2G by about 18-20% in all scenarios. Also in this case, the MESP-2G would not reach the MESP-1G even if the enzyme cost were set to zero.

**Figure 8:**
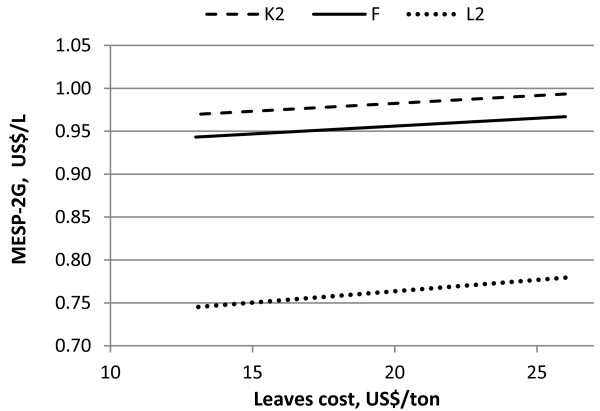
**Influence of leaves cost on the MESP-2G for Scenarios L, F and K2**.

The sugar cane accounts for a large proportion of the MESP of 1G ethanol production (61.3%). Nevertheless, changing this price has no effect on the MESP for 2G ethanol since it is calculated as a difference between the cost of 1G ethanol. Moreover, a change in the price of leaves would directly affect the MESP-2G, as can be seen in Figure 8. The cost of leaves contributes between 0.05 and 0.07 US$/L ethanol which, in the case of the lowest ethanol cost (Scenario L2), corresponds to about 9% of the overall MESP. If the cost of leaves is reduced by 50% the decrease in MESP-2G ranges from 2.4% to 4.5%.

**Figure 9:**
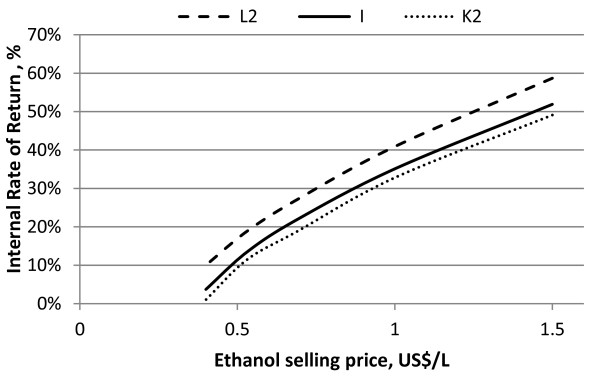
**Influence of ethanol selling price on the internal rate of return for Scenarios L2, I and K2**.

In Figure 9 the internal rate of return is calculated as function of the ethanol selling price and the trend curves are almost parallel for all three scenarios in spite of the different volumetric ethanol production. This suggests that the 2G ethanol volume produced is still not sufficient to achieve the profit breakeven with electricity revenues even at high ethanol selling prices (1.5 US$/L).

## Conclusions

Flowsheet models were developed for the design and simulation of various process layouts and for the assessment of the energy efficiency, capital and overall ethanol production costs in order to identify the process parameters and configurations having the lowest minimum ethanol selling price for 2G production.

Without subsidies, the MESP-2G from bagasse and leaves (0.99-0.78 US$/L) can compete with starch-based ethanol from Europe. This is to some extent dependent on the method used to calculate the cost of the 2G ethanol, which can thus be significantly influenced by the opportunity costs and revenues acting on the 1G ethanol production. Indeed a high sugar price can contribute to increased 1G ethanol costs reducing the gap between 1G ethanol and 2G ethanol production costs. On the contrary, higher revenues from electricity export can yield a wider gap, decreasing the competitiveness of 2G ethanol. In fact, after subtracting the income gained from the electricity sale, the minimum selling price for 1G ethanol is reduced by 30%, from 0.38 to 0.26 US$/L.

When the average ethanol selling price is 0.53 US$/L, the combined 1G + 2G ethanol production is economically feasible for all the cases simulated, even if the profitability is still lower (IRR < 21.5%) compared to 1G ethanol and electricity production (IRR = 32.1%). Higher yields in sugar recovery and conversion as well as a higher ethanol selling price might allow a possible profit breakeven of combined 1G + 2G ethanol with 1G ethanol.

Drying the EH solid residues to 80% DM in a steam dryer before CHP production reduced the 2G ethanol production cost, as more heat and power were produced from the same amount of DM, resulting in more bagasse being available for ethanol production.

It is important to obtain a high yield of fermentable sugars from the pretreatment and enzymatic hydrolysis steps. Doubling the enzyme dosage in the enzymatic hydrolysis can cause a lower MESP-2G, by about 12%, due to the increased ethanol production, of about 33%, as seen in Scenarios D vs. B. When leaves were used in addition to bagasse, the 2G ethanol production cost decreased in spite of the higher capital cost. The addition of leaves almost doubled the amount of 2G ethanol (compare Scenario F with Scenario D) as an effect of the higher sugar yield from leaves and the increase in electricity production due to more solids being available to the CHP plant. This resulted in a decrease in MESP-2G of almost 20%. However, when considering leaves, in the choice between "high WIS and lower yield in the EH" versus "low WIS and higher yield", the higher WIS pays off due to lower capital cost.

It is also fundamental to decrease the energy demand in the process or to increase the electricity production in the CHP plant as the electricity price has a considerable impact on the 2G ethanol production, since less electricity is exported compared to the Reference scenario, i.e. 1G ethanol production in an autonomous distillery. The most preferable way of reducing the energy demand is by heat integration wherever possible. Another way is by using higher WIS concentration in EH because of the higher ethanol concentration and lower energy demand in distillation. An increase in electricity production in the CHP plant can be achieved by drying the solids before burning, as shown in Scenario B. Another interesting option is biogas production from the stillage streams, although calculations suggest that batch anaerobic digestion (AD) is not cost effective due to the considerably diluted streams. This can be improved in the future by continuous AD, but requires further studies.

Finally, both the electricity price and the enzyme cost have considerable impact on the 2G ethanol production cost, as summarized in Table 5. A lower electricity price reduces the production cost of 2G ethanol, but the overall production cost, i.e. for both 1G and 2G ethanol, is negatively affected by the reduction in revenue from electricity export. A lower enzyme use cost could be achieved in several ways, e.g. by improving the pretreatment step, improving the efficiency of the enzymes or by reducing the production cost of the enzymes. Together with further pretreatment optimization, the more cost-effective enzyme products of the future (beyond the 2009 prototype used here) will release cost reduction potentials in the form of cheaper pretreatment, higher solids contents in enzymatic hydrolysis, higher yields and shorter residence times leading to cost reduction beyond that of the enzyme itself. Moreover, the fermentation of pentoses could considerably reduce the production cost of 2G ethanol, in particular when leaves are included in the feedstock. Only the future improvement of EH allowing operation with high solid contents has been considered in this study, so even though means of cost reduction have been identified in this work, there are still other options waiting to be explored to make 2G ethanol commercially feasible in the future.

**Table 5:** Main factors affecting positively and negatively (−) the MESP

Factors affecting MESP-2G	Factors affecting MESP-1G
Heat and process integration	Electricity opportunity cost

EH residues drying before combustion	Sugar opportunity cost (-) ^a^

Leaves addition	

High enzymes dosage	

Leaves EH at "high WIS, low yield"	

High WIS (16%)	

Electricity opportunity cost (-)	

Sugar opportunity cost ^a^	

^a ^*Not considered in this study*

## Method

The overall methodology used for technical and economic assessment can be divided in three main steps: flowsheet design and simulations, capital cost evaluation and investment cost analysis.

First, the flowsheets for the various process configurations were designed and simulated using the commercial software AspenPlus v7.1 (AspenTechnology Inc.) to perform rigorous thermodynamic calculations for mass and energy balances. Most of the process data implemented in AspenPlus were obtained experimentally within the project, such as bagasse and leaves composition [39], the recovery of sugars in the liquid and solid fractions after pretreatment, sugar yields from enzymatic hydrolysis at various process conditions, ethanol yield from fermentation and data for the combustion of solids residues [40]. Other data were estimated based on the authors' and partners' experience (running plant conditions).

The AspenPlus physical properties database contains most of the data needed for simulation of the entire process; but data for complex substances, for instance yeasts, enzymes and biomass components, such as lignin, were taken from the NREL physical properties database [41]. For specific process intermediates, such as bagasse and enzymatic hydrolysis (EH) residues, the overall higher heating value (HHV) obtained from the weighted sum of each structural component (e.g. lignin, cellulose) HHV embedded in the databases was in accordance with the experimentally determined values.

The output data from AspenPlus simulations were then used as input for the Aspen Process Economic Analyzer (AspenTechnology Inc.) for equipment sizing and for the estimates of both direct and indirect capital costs. For some specific equipment the cost was obtained from vendor quotations.

Finally, the overall ethanol production cost was calculated in a Microsoft^® ^Excel spreadsheet starting from the capital cost and the operating costs. The profitability of the various process configurations investigated was expressed as the minimum ethanol selling price for the 2G (cellulosic) ethanol (MESP-2G).

### Process description

Second-generation ethanol production from sugar cane residues can be positively influenced by integration with the 1G ethanol process and the benefits obtained depend on the level of process integration. Moreover, the operating conditions for the 2G process can also improve the overall economic feasibility. The aim of this study was to compare different operating conditions, process options and levels of integration for ethanol production from whole sugar cane in terms of energy efficiency and capital cost, in order to identify the lowest MESP.

A full-scale plant was modelled and the following four main processes were simulated: 1G ethanol production from sugar cane juice, 2G ethanol production from lignocellulosic residues of the sugar cane (bagasse and leaves), combined heat and power (CHP) plant and waste water treatment by anaerobic digestion (AD). The 1G process (autonomous distillery) comprises the traditional Melle-Boinot steps, well-experienced so far in the sugar cane-to-ethanol industry, while the 2G ethanol production is based on SHF of H_3_PO_4_-catalysed steam-pretreated bagasse and leaves. The CHP plant provides heat and power either to the 1G plant or to the combined 1G and 2G processes by burning bagasse, EH residues and biogas produced by AD.

Besides the process configurations, process variables found to have significant impact on the plant design and capital cost, based on experimental results obtained within the project, were investigated. These were mainly parameters associated with the enzymatic hydrolysis step, i.e. the residence time, enzyme dosage, concentration of water-insoluble solid material (WIS) in the pretreated material, as well as the addition of sugar cane leaves to raw material for 2G ethanol production. Among the pretreatment conditions tested experimentally for bagasse and leaves in the project, those resulting in the best EH yield were selected for model simulation.

### Autonomous distillery using sugar juice

The autonomous distillery modelled as it is today was used to represent the actual state of the art of an ethanol distillery using sugar cane juice in Brazil (1G), and was also considered as the initial flowsheet to which the 2G process was added (Figure 1). Data for this model were provided by Centro de Tecnologia Canavieira (CTC) - Piracicaba, SP, Brazil - for a conventional plant with an input flow rate of sugar cane equivalent to 540 tons sugar cane per hour (ton SC/h), or 165 tons dry sugar cane per hour (ton-dSC/h).

Sugar cane shredding and milling are the first processing stages, in which the juice used for fermentation in the 1G plant is separated from the bagasse, which is combusted in the CHP plant. The latter is used for the production of steam and electricity allowing the mill to be self-sufficient in energy, and providing excess electricity that can be sold. The sugar cane juice is purified by adding CaO and a flocculant polymer, followed by clarification. Since the sucrose concentration is too low (13.7 wt-%) to reach the desired ethanol titer in the fermentation stage, an evaporation unit is used to increase the concentration to 19 wt-%. The unit is composed of two evaporators in parallel, each with an area of 3500 m^2^, requiring 30 kg/h/m^2 ^live steam at 144°C and 2.5 bar from the CHP plant. The theoretical ethanol yield from fermentation based on the available sugars is 94%, and the ethanol concentration in the stream sent for distillation is 70 g/L. The secondary steam recovered after the evaporation flash is used to provide energy for distillation, using two strippers (34 trays each) and two rectifiers (56 trays each) arranged in parallel.

**Figure 10:**
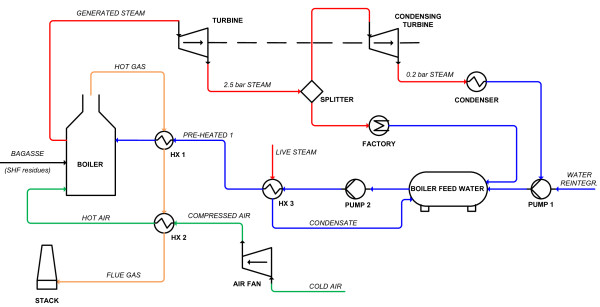
**Schematic flowsheet of the CHP plant**. Stream types are differentiated by colours: steam (red), condensate (blue), air for combustion (green), flue gases (orange).

Figure 10 shows the schematic flowsheet for the CHP plant. The boiler, having an efficiency of 0.80 based on the lower heating value, generates superheated steam at 500°C and 65 bar, which is used in turbines to supply electricity (77 MW_el_) and steam to the whole plant. The isentropic efficiency of the turbines was assumed to be 0.80. The steam for the plant is withdrawn at 144°C and 2.5 bar. In the autonomous distillery the material used in the CHP plant is the sugar cane bagasse at a dry matter (DM) content of 50% from the mill section. When bagasse is used in the 2G ethanol production process, the material combusted in the CHP plant consists predominantly of the solid residues after EH, mainly lignin, with the addition of some bagasse when the solid residues are not sufficient to meet the demand for steam from the 1G and 2G processes. When leaves are also used for ethanol production the material used in the CHP plant consists only of the solid residues after EH of bagasse and leaves. In the simulated scenarios, the boiler size was based on the mass flow feed rate of solids to the boiler, and the turbine system was modified to the steam requirement as the 2G process. Several turbine withdrawals were designed to supply superheated 20 bar steam to the dryer, saturated 10 bar steam for pretreatment, and 2.5 bar steam for evaporation and preheating of the feed to EH and distillation. A condensing turbine operating at 0.11 bar was also added when the steam requirement for the plant was lower than the total amount of steam produced.

### 2G ethanol from bagasse and leaves

Figure 2 shows the main steps involved in the 2G process investigated, consisting of steam pretreatment, solid-liquid separation, SHF and distillation, together with the 1G layout. The feedstock for the Base case consists of sugarcane bagasse from the mill section of the 1G plant at 50% DM, and is also the raw material in most of the other scenarios, except when leaves were added to the feedstock, in which case the amount of leaves is equivalent to 50% of bagasse on dry basis.

Bagasse (and leaves when added) are impregnated with H_3_PO_4 _(9.5 mg acid/g dry material) and then preheated to 95°C by direct injection of low-pressure secondary steam, before being fed to the continuous steam pretreatment reactor. Due to the high flow rate of feedstock two pretreatment reactors are needed for the bagasse. In the scenarios where leaves are also used a third pretreatment reactor is needed. The temperature in the reactor is maintained at 180°C by injecting high-pressure saturated steam at 10 bar, and the residence time is 10 minutes for both bagasse and leaves. Heat losses are assumed to be 10% of the adiabatic heat demand. The pretreated material is then flashed in three pressure reduction steps (7, 4, 1 bar), and the flash vapours obtained are condensed to heat other streams in the plant. After flashing part of the water in the slurry, the WIS concentration is 16%. The pretreatment step is the same in all scenarios.

The slurry from the last flash step in the pretreatment is neutralized to pH 5 with NH_3 _and washed in a filter press to recover the solubilized sugars, mainly pentoses, in the liquid fraction. The filter cake of solids content (35 wt-% DM) is diluted to obtain the desired WIS content, and enzymes are added for the EH process, which is performed at a temperature of 50°C. To investigate the effect of integrating the 1G and 2G processes, various residence times, enzyme loadings and WIS concentrations in the EH step were used. Yields of released glucose as a function of the enzyme dosage and residence time were based on experimental data, see Table 6. The residence time was set to 48 h or 72 h, depending on the scenario, and the size of the EH tanks was set to 1500 m^3^, resulting in 32 to 70 tanks. After EH, a filter press is used to separate the fermentable broth from the partially hydrolysed solid residues, which are sent to the CHP plant.

**Table 6:** Glucose released from enzymatic hydrolysis of bagasse and leaves at 7 % WIS ^a^

Enzyme dosage, reaction time	Glucose yield (%)	
	**Bagasse**	**Leaves**

Low, 48 h	42	60

High, 48 h	66	86

Low, 72 h	47	68

High, 72 h	73	96

^a ^*Unpublished from Knudsen BR - Novozymes Latin America Ldta*

The liquid is then fermented using an industrial yeast strain from CTC, giving 94% of the theoretical ethanol yield from hexose sugars. The complete cycle, which includes filling, fermentation, draining and cleaning, lasts 8.2 hours, and the size of the fermentors was set to 1200 m^3^, resulting in between 8 and 12 fermentors. The yeast is recovered by centrifugation prior to the liquid fraction entering the distillation unit, and low-pH washing of the yeast prevents any possible infections. The ethanol from the fermentation step is concentrated by distillation to 92.8 wt-%. The distillation section consists of two stripper columns (24 trays each), and a rectifier (36 trays), which are heat-integrated by operating at different pressures. The feed from the fermentation stage is divided between the two strippers. The first stripper is heated with live steam while the second stripper is heated by the overhead vapour from the first stripper. The rectifier is heated by the overhead vapour from the second stripper. Ethanol recovery was assumed to be 99.5% in each column.

The integrated 1G and 2G plant has many waste streams with a high COD content that can be treated by AD to produce biogas, which is burnt in the boiler for steam and electricity production. These streams include the bottom streams from distillation (stillage, also called vinasse), the condensates obtained during pretreatment, evaporation and drying, and the pentose-rich liquid fraction from filtration of the pretreated material prior to enzymatic hydrolysis. Experimental trials were performed to obtain data for the calculation of the biogas potential from these streams. These data are required as input to the flowsheet calculations and are fundamental in the energy balance of the integrated 1G and 2G system, since the amount of biogas can be important in improving the energy supply and reducing the final ethanol production cost. The biogas potential was measured in batch trials for several stillage streams and for the pentose-rich stream after filtration of the pretreated material (data not shown).

The microorganism consortium was taken from the Domsjö Fabriker AB spent sulphite liquor plant (Domsjö, Sweden). The stillage streams required at least 10 days residence time in AD, whereas the pentose-rich samples reached maximum production after about 5 days. Continuous AD could probably shorten the residence time, but the results for the batch system were used to be on the conservative side. The final methane yield was 0.112 and 0.127 g_CH4_/g_COD _for the stillage and pentose-rich streams from bagasse, respectively. Table 7 presents a summary of the streams that can be used in AD in the combined 1G + 2G plant. All the streams are not suitable for anaerobic digestion, due to high flow rate or high dilution of COD, which means a high capital cost compared with the methane production capacity. Indeed, if all the streams were subjected to AD, 151 stirred tank reactors with a volume of 2500 m^3 ^each would be required, and the investment cost for the biogas facility alone would be about 68 million US$. For this reason, only AD of the pentose-rich stream, which has a higher content of COD and requires 98 hours residence time to reach 96% of the maximum biogas production with a yield of 0.116 g_CH4_/g_COD_, was included in the final simulations.

**Table 7:** Origin and composition of streams used in anaerobic digestion

Stream origin	Flow rate (ton/h)	Water content (%)
Pentoses from SHF	217	91.5

Pretreatment condensate	28	95.0

Strippers 1G	490	98.0

Rectifiers 1G	19	99.0

Strippers 2G	642	99.0

Rectifier 2G	35	99.9

### The integrated 1G + 2G plant

When the 2G plant is simply annexed to the existing 1G facility, there is no integration except the sharing of heat and power produced in the CHP plant. Thus the synergies arising from the combination of the two processes cannot be exploited to minimize the energy demand and the capital cost. Alternative configurations for the combined 1G + 2G facility rely on the strategy adopted for integrating the 1G and 2G processes in a new plant. For this reason, energy reduction and recovery, as well as process stream mixing are the integration options that were investigated to find the configuration with the lowest MESP-2G. Energy integration is achieved by further increasing the use of new and more efficient equipment, suitable for recovery and/or in a different configuration. However, new equipment can significantly increase the capital investment cost, thus the economic feasibility of better energy use was also evaluated. Process energy efficiency was defined as the energy output in the products (ethanol and excess electricity) divided by the energy input, i.e. the energy contained in the raw materials (sugar cane, and leaves in the scenarios were these were included). Calculations were based on the HHVs of the raw materials and ethanol. The electric power was recalculated in terms of the fuel necessary to produce this electricity, assuming an electricity-to-fuel ratio of 0.33.

Regarding stream integration, mixing of material flows can have beneficial effects on the energy balance, especially in the distillation and evaporation stages. Two mixing options were considered in an attempt to reduce the energy demand in these two steps: mixing of the 1G ethanol-rich stream and the 2G ethanol-poor stream prior to the distillation unit (see Figure 3); and mixing of the 2G glucose stream after EH with the 1G sugar juice prior to and after evaporation (see Figure 4).

### Economic calculations

The results from AspenPlus simulations (mass and energy balances) were imported into Aspen Process Economic Analyzer v.7.1 (Aspen Technology, Inc.) where equipment was sized and direct/indirect costs were determined. Fixed capital investment costs were estimated based on costs for the first quarter of 2008, except for the cost of the pretreatment unit, which was obtained from a vendor quotation based on pretreatment using SO_2 _as acid catalyst (in 2010). The fixed capital investment cost was recalculated from 2008 to 2010 using cost indices for Houston, Texas, USA. A factor of 0.82 was then applied to scale fixed capital investment costs from the US gulf coast to Brazilian conditions. These data, together with stream data, were exported from Aspen Process Economic Analyzer and used in the Excel model developed for the final cost calculations.

The Excel model comprises investment costs and data on biomass, chemicals, enzymes, electricity price and all other running costs. The main premises for the production cost estimate are that it is a stand-alone ethanol production plant processing 165 dry metric ton of sugar cane per hour and is operated 200 days per year. The plant is equipped with a CHP plant for production from residue and/or sugar cane leaves. Excess electricity is exported.

It is important to point out that electricity is accounted for as an opportunity cost; negative for the 1G process (revenue from selling electricity produced by burning bagasse) and positive for the 2G plant (cost due to the increased energy demand compared with the 1G process alone). The plant is assumed to be the N^th ^plant, meaning that it is considered to be based on known technology at the time of construction (N could be 3-6 depending on the size and complexity of the demonstration plant). The Minimum Ethanol Selling Prices (MESPs) for the integrated 1G + 2G process and the separate processes are calculated as the sum of each single production cost item (see Table 3 and Table 4). The marginal cost items accounted for the 2G process are derived from the following expression in respect to the integrated 1G + 2G process and 1G process.

(1) $${P^{i}}_{2G}=\frac{F_{1G+2G}\cdot {P^{i}}_{1G+2G}-F_{1G}\cdot {P^{i}}_{1G}}{F_{1G+2G}-F_{1G}}$$

where *P^i ^_2G _*is the 2G cellulosic ethanol production cost for the item *i *given by a weighted ratio between the difference in the cost of item *i *for 1G + 2G and 1G ethanol and the volume of 2G ethanol produced. The data for 1G ethanol production, indicated by the index *1G*, were derived for the autonomous distillery (Reference scenario). *P^i ^*denotes the production cost for item *i *and *F *the amount of ethanol produced in the specific process. The MESP is not only a measure of total production cost but also includes the investment return, accounted for as capital cost, at the desired internal rate of return (IRR). The MESP was varied in the spreadsheet model until the net present value (NPV) equalled to zero at the selected IRR (10%). The NPV was calculated based on the values given in Table 8. The prices of all raw materials and products were assumed to be subject to the same rate of inflation. Tax-deductible depreciation was assumed to be 20% p.a. for fixtures, cars and IT, 10% for process equipment and 4% for buildings. Since the largest cost is that of process equipment, 10% was chosen as an average for simplicity, i.e. linear full depreciation in ten years. The cost of sugarcane was assumed to be 65 US$/dry ton delivered to the plant, based on whole unwashed stalks and the DM content was assumed to be 30%. The cost of sugar cane leaves was assumed to be 26 US$/dry ton delivered to the plant, unground and unwashed and the DM content was assumed to be 50%. Costs for chemicals are given in Table 9. The enzyme cost is based on budget price for the cutting-edge proteins in 2009. The electricity selling price (daily and annual average) to the local grid was assumed to be 87 US$/MWh. The value of the treated water was set to 0.03 US$/ton, and the stillage stream, which is recycled to fields, was assumed to have a value of 1.6 US$/ton based on the N, P and K in the stream. Labour costs were assumed to be 3.5 million US$ per year.

**Table 8:** Main assumptions for economic calculations

Parameter	Value
Internal rate of return after tax and above inflation	10%

NPV duration	20 years

Tax rate	34%

Tax-deductible linear depreciation for capital cost	10 years

Plant scrap value	None

Payment of total project investment ahead of start-up	12 months

Working capital (% of turnover)	20%

Financing	100% equity

Currency basis	2010 US$

**Table 9:** Cost of chemicals and water

Chemicals	**Cost **(US$/ton)
Sulphuric acid (concentrated H_2_SO_4_)	100

Phosphoric acid (as 50 wt-% acid)	500

Ammonia (as 25 wt-% NH_3_)	100

NaOH (as 50 wt-% NaOH)	200

Enzyme product (NS22086)	Budget price 2009

(NH_4_)_2_HPO_4_	1000

MgSO_4_	3000

Cooling water	0.08

Process water	0.33

## Endnotes

^# ^European Commission-funded project within the 7^th ^Framework Programme in partnership with Brazil. Period March 2009 - February 2011. Project participants: Centro de Tecnologia Canavieira, Piracicaba, SP, Brazil; Universidade Federal do Paranà, PR, Brazil; Novozymes Latin America, Brazil; Novozymes Denmark; and Lund University, Sweden.

## Abbreviations

1G: first generation; 2G: second generation; 1G + 2G: combined first and second generation; AD: anaerobic digestion; CHP: combined heat and power; DM: dry matter; dSC: dry sugar cane; EH: enzymatic hydrolysis; MESP: minimum ethanol selling price; SC: sugar cane; SHF: separate hydrolysis and fermentation; SSF: saccharification and simultaneous fermentation; WIS: water insoluble solids

## Competing interests

The authors declare that they have no competing interests.

## Authors' contributions

SM carried out the simulations and economic evaluation and wrote the paper. JM developed the model for financial calculations. GZ, JM and SM designed and analysed the study. GZ and JM reviewed the paper. All authors read and approved the final manuscript.
